# Success rates of re-excision after positive margins for invasive lobular carcinoma of the breast

**DOI:** 10.1038/s41523-019-0125-7

**Published:** 2019-09-06

**Authors:** Merisa L. Piper, Jasmine Wong, Kelly Fahrner-Scott, Cheryl Ewing, Michael Alvarado, Laura J. Esserman, Rita A. Mukhtar

**Affiliations:** 1 Division of Plastic Surgery, Department of Surgery, University of California, San Francisco, CA USA; 2 Division of General Surgery, Department of Surgery, UCSF Helen Diller Family Comprehensive Cancer Center, University of California, San Francisco, CA USA

**Keywords:** Breast cancer, Cancer therapy, Outcomes research

## Abstract

Rates of positive margins after surgical resection of invasive lobular carcinoma (ILC) are high (ranging from 18 to 60%), yet the efficacy of re-excision lumpReceptor subtypeectomy for clearing positive margins is unknown. Concerns about the diffuse nature of ILC may drive increased rates of completion mastectomy to treat positive margins, thus lowering breast conservation rates. We therefore determined the success rate of re-excision lumpectomy in women with ILC and positive margins after surgical resection. We identified 314 cases of stage I-III ILC treated with breast conserving surgery (BCS) at the University of California, San Francisco. Surgical procedures, pathology reports, and outcomes were analyzed using univariate and multivariate statistics and Cox-proportional hazards models. We evaluated outcomes before and after the year 2014, when new margin management consensus guidelines were published. Positive initial margins occurred in 118 (37.6%) cases. Of these, 62 (52.5%) underwent re-excision lumpectomy, which cleared the margin in 74.2%. On multivariate analysis, node negativity was significantly associated with successful re-excision (odds ratio [OR] 3.99, 95% CI 1.15–13.81, *p* = 0.029). After 2014, we saw fewer initial positive margins (42.7% versus 25.5%, *p* = 0.009), second surgeries (54.6% versus 20.2%, *p* < 0.001), and completion mastectomies (27.7% versus 4.5%, *p* < 0.001). In this large cohort of women with ILC, re-excision lumpectomy was highly successful at clearing positive margins. Additionally, positive margins and completion mastectomy rates significantly decreased over time. These findings highlight improvements in management of ILC, and suggest that completion mastectomy may not be required for those with positive margins after initial BCS.

## Introduction

Obtaining clear margins after surgical resection of breast cancer is a well-documented challenge in the management of this disease, and having unresected positive margins is associated with worse outcomes.^1–3^ For invasive lobular carcinoma (ILC), the second most common subtype of breast cancer, the issue of positive margins is a particularly prevalent problem. ILC lacks the adhesion protein E-cadherin, resulting in a diffuse pattern of tumor growth in so-called “single file” lines of tumors cells. Additionally, imaging tests often underestimate tumor size in ILC. The combination of a diffuse growth pattern and high false negative rates on imaging results in higher rates of positive margins compared to invasive ductal carcinoma (IDC).^4,5^ Indeed, up to 60% of women with ILC who undergo breast conservation surgery (BCS) will have positive margins.^2,6–12^

For the large number of women who have positive margins after partial mastectomy for ILC, they and their physicians must decide whether to pursue re-excision in a continued attempt to conserve the breast, versus completion mastectomy. Concerns that re-excision lumpectomy will fail to clear the margin can result in increased mastectomies, and in several series, women with ILC who have positive or close margins are significantly more likely to undergo completion mastectomy than women with IDC.^2,12–15^ While many investigators have reported on high positive margin rates in ILC, to our knowledge there are scarce data reporting the success rates of re-excision lumpectomy at clearing the initially positive margin. Understanding the likelihood of success is critical for patients to make informed decisions about whether to continue to pursue breast conservation after an initial positive margin, versus undergoing completion mastectomy.

Our primary goal, therefore, was to determine the success rate of re-excision lumpectomy for positive margins after partial mastectomy for ILC. Our secondary goals were to identify factors associated with successful re-excision lumpectomy, to determine the impact of persistently positive margins on disease free survival (DFS), and lastly, to evaluate changes in the incidence and management of positive margins before and after margin consensus guidelines of 2014.

## Results

### Patient characteristics

Average age at diagnosis was 61.8 years (range 30–97), 93.2% of cases were estrogen receptor (ER) positive/human epidermal growth factor receptor 2 (HER2) negative, and 62.0% of cases were grade 2. The majority of patients had stage 1–2 disease, and average follow up time was 6.1 years, ranging from 0.5 months to 26 years (Table 1).

**Table 1:** Patient and tumor characteristics

Characteristic	*N* (%)
Age, years [median (range)]	61.3 (30–97)
Post-menopausal^a^	181 (73.6%)
Tumor grade^b^	
1	103 (33.4%)
2	191 (62.0%)
3	14 (4.6%)
Receptor subtype^c^	
ER+/PR+/HER2−	216 (78.3%)
ER+/PR−/HER2−	41 (14.9%)
HER2+	14 (5.1%)
Triple negative	5 (1.8%)
Lymphovascular invasion	18 (5.7%)
Presence of lobular carcinoma in situ^d^	232 (75.1%)
Tumor multifocality	97 (31.3%)
Pleomorphic ILC	31 (9.9%)
Nodal stage	
N0	239 (76.1%)
N1	47 (15.0%)
N2	11 (3.5%)
N3	17 (5.4%)
Tumor stage	
T1	194 (61.8%)
T2	96 (30.6%)
T3	24 (7.6%)
Overall stage	
1	244 (77.7%)
2	50 (15.9%)
3	20 (6.4%)
Follow-up time, years [median (range)]	4.5 (0.5–26)

Data are expressed as *n* (%) unless otherwise specified

*ER* estrogen receptor, *PR* progesterone receptor, *HER2* human epidermal growth factor receptor 2

^a^Data available in *n* = 246; ^b^data available in *n* = 308; ^c^data available in *n* = 276; ^d^data available in *n* = 309

### Success rate of re-excisions for positive margins

**Fig. 1:**
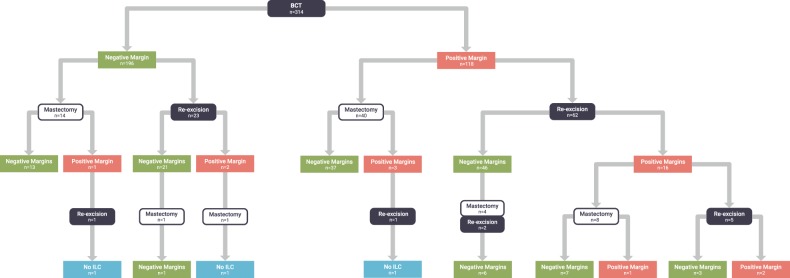
Flow chart of outcomes of patients with ILC who initially underwent BCS

In our cohort of 314 ILC cases treated with BCS, positive margins occurred in 118 (37.6%). Of these, 102 cases had additional surgery, of which 62 were re-excision lumpectomies and the remainder completion mastectomies. Among the 62 re-excision lumpectomies for positive margins, 46 (74.2%) were successful, meaning they resulted in negative margins, while 16 cases resulted in positive margins again. Of these 16 cases with positive margins after first attempt at re-excision, 8 (50%) underwent completion mastectomy, 5 (31.3%) had a second re-excision lumpectomy, and 3 (18.8%) had no further surgery (Fig. 1).

Among the 62 cases who had re-excision lumpectomies for positive margins, we compared the successful re-excisions (*n* = 46) to the unsuccessful re-excisions (*n* = 16) to identify factors associated with higher likelihood of success. Between these groups, there was no difference in rates of pleomorphic ILC, lymphovascular invasion, tumor subtype by ER/PR/HER2 status, grade, presence of lobular carcinoma in situ, or tumor multifocality (Table 2). Mean tumor size was larger in those with unsuccessful re-excision (3.1 cm versus 2.4 cm), but this difference did not reach statistical significance. However, those with successful re-excision had significantly less nodal involvement than those with unsuccessful re-excision (mean of 1.3 positive nodes versus 4.6 positive nodes, *p* = 0.0298). Additionally, older women were significantly more likely to have a successful re-excision lumpectomy (mean age 61.4 versus 55.1 years in successful versus unsuccessful cases, *p* = 0.0445). In a logistic regression model adjusting for age and nodal status, node negativity remained the only significant predictor of successful re-excision (OR 3.99, 95% CI 1.15–13.81, *p* = 0.029).

**Table 2:** Factors associated with successful re-excision lumpectomy

	Successful re-excision lumpectomy (*n* = 46)	Unsuccessful re-excision (*n* = 16)	*p*-value
Age in years (mean, median, [range])	61.4, 60.7 (43.7–80.4)	55.1, 52.6 (44.7–78.8)	0.0445
Post menopausal	25 (71.4%)	6 (54.6%)	NS
Tumor grade (*n* = 61)			
1	16 (35.6%)	7 (43.8%)	NS
2	27 (60%)	9 (56.3%)	
3	2 (4.4%)	0 (0%)	
Receptor subtype			
ER+/PR+/HER2−	33 (78.6%)	11 (73.3%)	NS
ER+/PR−/HER2−	8 (19.1%)	3 (20%)	
HER2+	1 (2.4%)	1 (6.7%)	
Lymphovascular invasion	7 (15.2%)	1 (6.3%)	NS
Presence of LCIS	37 (82.2%)	14 (87.5%)	NS
Tumor multifocality	21 (46.7%)	9 (60%)	NS
Pleomorphic	4 (8.7%)	1 (6.3%)	NS
Lymph node positive	12 (26.1%)	10 (62.5%)	0.009
N stage			0.029
0	34 (73.9%)	6 (37.5%)	
1	8 (17.4%)	5 (31.3%)	
2	1 (2.2%)	2 (12.5%)	
3	3 (6.5%)	3 (18.8%)	
Mean tumor size (cm, SD)	2.4, 1.6	3.1, 2.3	NS
T stage			NS
1	27 (58.7%)	7 (43.8%)	
2	15 (32.6%)	4 (25%)	
3	4 (8.7%)	5 (31.3%)	
Era of treatment			NS
Before 2014	36 (73.5%)	13 (26.5%)	
2014–2018	10 (76.9%)	3 (23%)	

*NS* not significant, *SD* standard deviation

### Re-excisions for negative margins

There were patients who underwent a second surgery despite having negative margins (defined as no ink on tumor) after initial BCS. Among the 196 patients with negative margins, 37 (18.9%) underwent re-excision (either re-excision lumpectomy or completion mastectomy, Fig. 1). Of the 175 negative margin cases with margin width available, the average margin width was significantly smaller among those who underwent second surgery compared to those who did not undergo a second surgery (1.27 mm versus 2.37 mm, respectively, *p* = 0.0118).

### Unresected positive margins

Among the entire cohort of 314 ILC cases, 175 (55.7%) had a single surgery, 117 (37.3%) had two surgeries, and 22 (7%) had 3 surgeries. BCS was successful in 246 (78.3%), with the remaining cases undergoing completion mastectomy. Ultimately, 288 (91.7%) had negative margins, while 26 (8.3%) had unresected positive margins (4 of which occurred despite completion mastectomy). Of the 26 unresected positive margins, 16 (61.5%) were either anterior or posterior, 8 (30.7%) were radial margins (either superior, medial, lateral, or inferior), and 2 were of unknown location. All unresected positive radial margins occurred in BCS cases with no completion mastectomy performed. On univariate Cox proportional hazards analysis, having a final positive margin was significantly associated with shorter DFS (HR 3.4, 95% CI 1.3–8.9, *p* = 0.014). However, when adjusting for age, stage, tumor subtype, tumor grade, local therapy, and adjuvant chemotherapy use, final margin status was no longer significantly associated with DFS (HR 3.4, 95% CI 0.8–13.9, *p* = 0.087), while tumor grade (HR 10.3, 95% CI 1.13–94.3, *p* = 0.039) and subtype (HR 6.1, 95% CI 1.3–27.5, *p* = 0.02) remained significant predictors of DFS (Table 3).

**Table 3:** Cox proportional hazards model for DFS

	HR	*p*-value	95% CI
Age at diagnosis	0.97	0.247	0.92–1.02
Stage			
1	Reference		
2	0.37	0.361	0.045–3.08
3	0.62	0.664	0.072–5.35
Receptor subtype			
ER+/PR+/HER2–	Reference		
ER+/PR-/HER2–	6.1	0.020	1.33–27.47
HER2+	0	N/A	N/A
Triple negative	0	N/A	N/A
Grade			
Low/intermediate	Reference		
High	10.3	0.039	1.13–94.28
Final positive margins	3.41	0.087	0.84–13.89
Local therapy			
Lumpectomy with radiation	Reference		
Lumpectomy alone	0.78	0.769	0.15–4.0
Mastectomy	1.8	0.371	0.49–6.62
Mastectomy with radiation	0	N/A	N/A
Adjuvant chemotherapy	0.8	0.725	0.22–2.83

*DFS* disease-free survival, *HR* hazard ratio, *CI* confidence interval, *N/A* not applicable

### Era of treatment

Finally, we evaluated the impact of era of treatment before and after 1 January 2014. While the definition of adequate margins in clinical practice has varied over time, in 2014 Society of Surgical Oncology (SSO)/American Society for Radiation Oncology (ASTRO) consensus guidelines defined adequate margins as no ink on tumor.^16^ In our cohort, positive margin rates, re-excision rates for both positive and negative margins, and completion mastectomy rates were significantly higher prior to 2014 (Table 4). We specifically evaluated rates of re-excision for margin width ≤1 mm, and found significantly higher re-excision rates prior to 2014 (50% versus 15.6%, *p* = 0.001). Of the 37 re-excisions performed for negative margins (no ink on tumor), 35 (94.6%) were performed prior to 2014.

**Table 4:** Margin status and management before and after consensus guidelines of 2014

	Overall	Before 2014	2014–2018	*p*-value
Final margin width, mm (mean, median, SD)	2.55, 1.5, SD 2.94	2.64, 1.5, SD 3.17	2.34, 1, SD 2.37	0.4978
Positive margins at initial BCS (defined as ink on tumor)	118 (37.6%)	94 (42.7%)	24 (25.5%)	0.009
Cases undergoing re-excision lumpectomy or completion mastectomy for any indication	139 (44.3%)	120 (54.6%)	19 (20.2%)	<0.001
Cases undergoing re-excision lumpectomy or completion mastectomy for positive margins	102 (86.4%)	85 (90.4%)	17 (70.8%)	0.012
Cases undergoing re-excision lumpectomy or completion mastectomy for negative margins	37 (18.9%)	35 (27.8%)	2 (2.9%)	<0.001
Completion mastectomy rate	68 (21.7%)	61 (27.7%)	7 (4.5%)	<0.001

## Discussion

In this analysis of outcomes for a large cohort of women with ILC undergoing BCS, we found that when re-excision lumpectomy was attempted to clear positive margins, it was successful 74.2% of the time (46 out of 62 re-excision lumpectomies). Those with smaller tumors, older age, and lower burden of nodal involvement had the highest rates of successful re-excision lumpectomy. However, the only significant predictor of successful re-excision lumpectomy was node negative status (which the majority [76.1%] of these patients had). Currently, women with ILC have high rates of completion mastectomies in the setting of positive margins following attempted BCS. An institutional series of over 10,000 breast cancer patients undergoing BCS found that 62.7% of the 1215 ILC patients had a completion mastectomy.^17^ Our data suggest that for women with ILC who have positive margins after BCS, attempting a re-excision lumpectomy is reasonable and likely to be successful. These high rates of completion mastectomy may not be necessary in all women with ILC who have positive margins.

Achieving a clear margin, however, is still an important goal in the surgical management of ILC. On univariate analysis, those patients with persistently positive margins had significantly worse DFS. The impact of positive margins was mitigated when adjusting for other factors on multivariate analysis, but the trend towards decreased DFS remained. Whether or not margin width impacts DFS was beyond the scope of this analysis, but the significant change in margin management over time seen in our cohort will require ongoing study to evaluate long term outcomes. Similar to other reports, we found that after publication of SSO/ASTRO consensus guidelines in 2014, the rate of re-excision for close margins was markedly reduced.^18^ This appeared to translate into far fewer completion mastectomies (27.7% completion mastectomy rate prior to 2014, compared to 4.5% rate after 2014, *p* < 0.001). Simultaneous with this change was a significant reduction in initial positive margin rate. This may reflect the incorporation of surgical techniques such as use of shave margins and oncoplastic surgery, which have been shown to reduce positive margin rates in ILC.^15^ Other tools to reduce positive margin rates include intraoperative margin assessment, with some centers reporting positive margin rates as low as 3.6% when frozen section is utilized, and potential neoadjuvant approaches to downstage tumors.^19,20^ Because ILC tends to have late recurrences, further study will be needed to evaluate the impact of these management changes in ILC specifically.^21^

While the initial positive margin rate of 37.6% after BCS in our cohort falls within the reported range for ILC, it is still quite high. While some centers advocate excluding patients at higher risk for positive margins from undergoing BCS (e.g., those with T3 tumors or multifocality)^22^ we do not routinely exclude these patients from attempting BCS provided that they understand the associated risks and make an informed decision to proceed. This inclusion of higher risk patients may contribute to high positive margin rates.

This study has several strengths, including the careful review of outcomes on multiple surgical procedures, long follow up time, and the applicability to patients since these findings reflect standard clinical care decisions outside the context of a clinical trial. However, one major limitation of retrospective analyses is the inability to determine which factors drove re-excisions. For example, some patients may have elected to undergo re-excision or mastectomy because of personal preference, and some positive margins may have been left unresected due to a surgeon’s impression that no breast tissue remained for excision. This is supported by the finding that the majority of unresected positive margins were located at the anterior or posterior location, and standard oncologic resections go from dermis to muscle at our institution.

These findings provide an in depth analysis of the success rates of re-excision lumpectomy after the finding of initial positive margins in ILC, and can be used to help patients make informed surgical choices. Given a high likelihood of success, attempting re-excision lumpectomy is reasonable, and, is even more likely to be successful in patients without nodal involvement. While undergoing any additional surgery is associated with patient morbidity and potential delay in starting adjuvant therapy, a population based study of over 11,000 patients with breast cancer found no survival difference between patients with positive margins who underwent continued attempt at BCS compared to completion mastectomy.^23^

In summary, our report on the success rate of re-excision lumpectomy for patients with ILC should provide guidance to both patients and surgeons in making management recommendations in the all too common scenario of finding positive margins. Although BCS can be successful in many women with ILC, more work is needed to identify therapeutic approaches that reduce tumor size and result in lower rates of positive margins for ILC, and longer term follow-up on the impact of changes in margin management is necessary.

## Methods

### Cohort description

We queried a prospectively maintained surgical database and the pathology archives at the University of California, San Francisco to identify patients with the diagnosis of ILC. We identified 675 cases of ILC treated between 1992 and June 2018. After excluding those with missing surgical treatment data, de novo stage 4 disease, those missing data for margin status at first or second excision, those undergoing initial mastectomy, and those receiving neoadjuvant therapy, we included 314 cases in the analysis. We collected data on patient demographics, operative details involving the initial and all subsequent breast cancer operations, pathology findings, and outcomes including time to local or distant recurrence. Disease free survival was defined as time from cancer diagnosis to first recurrence, whether ipsilateral locoregional, or distant; patients were censored at the time of first recurrence. This study was approved by the Institutional Review Board at the University of California, San Francisco; informed consent was not required given no patient contact was needed for this study.

Surgical margin status and width in 1 mm increments were recorded for all surgical procedures when available. Positive margins were defined as ink on tumor, as described in clinical pathology reports, based on the guidelines published by the Society of Surgical Oncology (SSO) and the American Society for Radiation Oncology (ASTRO) in 2014.^16^ Successful BCS was defined as the absence of undergoing mastectomy. Successful re-excision was defined as a re-excision lumpectomy that resulted in negative margins. Pathologic staging was assigned according to the American Joint Committee on Cancer 7th edition.^24^

### Statistical methods

We analyzed the data in Stata 14.2. We used Chi-squared tests and Fisher’s exact test for categorical variables, the Wilcoxon rank-sum test for continuous variables, logistic regression, and Cox proportional hazards models. Two-tailed *p* values < 0.05 were considered significant.

### Reporting summary

Further information on research design is available in the Nature Research Reporting Summary.

## Supplementary information

Reporting Summary Checklist

## Data Availability

The data generated and analyzed during this study are described in the following data record: 10.6084/m9.figshare.9578885.^25^ The data supporting all four tables in this published article are not publicly available to protect patient privacy, but can be accessed from the corresponding author on request, as described in the data record above. Data will be made available to authorized researchers who have obtained institutional review board (IRB) approval from their own institution and from the UCSF IRB.

## Supplementary information

**Supplementary Information** accompanies the paper on the *npj Breast Cancer* website (10.1038/s41523-019-0125-7).

## Code availability

Stata 14.2 code used for data analysis are available upon request.
